# HDAC1 Controls CD8^+^ T Cell Homeostasis and Antiviral Response

**DOI:** 10.1371/journal.pone.0110576

**Published:** 2014-10-21

**Authors:** Roland Tschismarov, Sonja Firner, Cristina Gil-Cruz, Lisa Göschl, Nicole Boucheron, Günter Steiner, Patrick Matthias, Christian Seiser, Burkhard Ludewig, Wilfried Ellmeier

**Affiliations:** 1 Division of Immunobiology, Institute of Immunology, Center for Pathophysiology, Infectiology and Immunology, Medical University of Vienna, Vienna, Austria; 2 Institute of Immunobiology, Cantonal Hospital St.Gallen, St. Gallen, Switzerland; 3 Division of Rheumatology, Medicine III, Medical University of Vienna, Vienna, Austria; 4 Friedrich Miescher Institute for Biomedical Research, Novartis Research Foundation and University of Basel, Faculty of Sciences, Basel, Switzerland; 5 Department of Medical Biochemistry, Max F. Perutz Laboratories, Vienna Biocenter, Medical University of Vienna, Vienna, Austria; Federal University of São Paulo, Brazil

## Abstract

Reversible lysine acetylation plays an important role in the regulation of T cell responses. HDAC1 has been shown to control peripheral T helper cells, however the role of HDAC1 in CD8^+^ T cell function remains elusive. By using conditional gene targeting approaches, we show that *LckCre*-mediated deletion of HDAC1 led to reduced numbers of thymocytes as well as peripheral T cells, and to an increased fraction of CD8^+^CD4^–^ cells within the CD3/TCRβ^lo^ population, indicating that HDAC1 is essential for the efficient progression of immature CD8^+^CD4^–^ cells to the DP stage. Moreover, CD44^hi^ effector CD8^+^ T cells were enhanced in mice with a T cell-specific deletion of HDAC1 under homeostatic conditions and HDAC1-deficient CD44^hi^ CD8^+^ T cells produced more IFNγ upon *ex vivo* PMA/ionomycin stimulation in comparison to wild-type cells. Naïve (CD44^l^°CD62L^+^) HDAC1-null CD8^+^ T cells displayed a normal proliferative response, produced similar amounts of IL-2 and TNFα, slightly enhanced amounts of IFNγ, and their *in vivo* cytotoxicity was normal in the absence of HDAC1. However, T cell-specific loss of HDAC1 led to a reduced anti-viral CD8^+^ T cell response upon LCMV infection and impaired expansion of virus-specific CD8^+^ T cells. Taken together, our data indicate that HDAC1 is required for the efficient generation of thymocytes and peripheral T cells, for proper CD8^+^ T cell homeostasis and for an efficient *in vivo* expansion and activation of CD8^+^ T cells in response to LCMV infection.

## Introduction

Dynamic changes in histone acetylation patterns are mediated by the activity of histone acetyltransfereases (HATs) and histone deacetylases (HDACs) and are key events in the epigenetic regulation of gene expression. In addition, many non-histone targets of HATs/HDACs have been described and it has been demonstrated that reversible lysine acetylation can affect protein-protein and protein-DNA interactions, protein stability and intracellular localization. This implies that lysine acetylation is an important post-translational modification regulating a variety of cellular pathways and thus broadening the functional role of HATs/HDACs beyond epigenetic gene regulation [1].

The application of HDAC inhibitors revealed a variety of T cell functions controlled by reversible lysine acetylation [2]. The mammalian HDAC family is sub-divided into 4 classes consisting of 18 members [3] and several HDAC family members have been implicated in the regulation of T cell development and differentiation [2], [4]. The combined activity of HDAC1 and HDAC2 is essential for the progression of double-negative (DN) to double-positive (DP) thymocytes [5], [6]. HDAC7 regulates both positive and negative selection during T cell development [7]–[9] and class II HDACs (HDAC4, 5 and 10) have been implicated in the ThPOK-mediated silencing of the *Cd8* gene loci during CD4 lineage differentiation [10]. HDACs have also been connected to the regulation of regulatory T cell function [11]. The activity of FoxP3 is regulated by acetylation [12] and it has been shown that HDAC7 and HDAC9 bind to FoxP3. This suggests that both HDAC7 and HDAC9 might regulate the activity of FoxP3 and Tregs. Moreover, HDAC6- or HDAC9-deficiency leads to increased Treg numbers and enhanced Treg function [13], [14]. HDAC7 also controls CTL function, since HDAC7 function has been linked with the repression of key cytokines, cytokine receptors and adhesion molecules important for CTL function [15]. Further, it has also been shown that HDAC1 and HDAC2 are essential to prevent neoplastic transformation of immature T cells [5], [6]. By using conditional gene targeting approaches, we previously showed that HDAC1 is a key regulator of Th2 cytokine responses [16]. Loss of HDAC1 (using the *Cd4Cre* delete strain) led to an increased inflammatory response in an *in vivo* allergic airway inflammation model and mice with HDAC1-deficient T cells displayed an increase in all clinical parameters of this Th2-type asthma model. This correlated with enhanced Th2 cytokine production of HDAC1-deficient T cells isolated from diseased mice. Although this study clearly demonstrated an important function for HDAC1 in peripheral T helper cells, the role of HDAC1 in CD8^+^ T cells as well as during earlier steps of T cell development has not been explored.

In this study we employed conditional gene targeting approaches to investigate the role of *Hdac1* during early T cell development using the *LckCre* deleter strain. Moreover, we studied whether CD8^+^ T cell function and effector differentiation are regulated by HDAC1 under steady state conditions and during viral infection using *Cd4Cre*-mediated deletion of HDAC1. Although the relative distribution of DP and mature CD4SP and CD8SP cells as well as the expression of surface marker such as CD24, CD5 and CD69 was not changed, *LckCre*-mediated loss of HDAC1 led to reduced numbers of thymocytes as well as peripheral T cells. There was an increased fraction of CD8^+^CD4^–^ cells within the CD3/TCRβ^lo^ population in *Hdac1^f/f^LckCre* mice, indicating that HDAC1 is essential for the efficient progression of immature CD8SP cells to the DP stage. In addition, we observed that CD44^hi^ effector CD8^+^ T cells were enhanced in mice with a T cell-specific loss of HDAC1 under homeostatic condition and that *Hdac1^f/f^LckCre* CD44^hi^ CD8^+^ T cells produced more IFNγ upon *ex vivo* PMA/ionomycin stimulation in comparison to wild-type cells. Naïve (CD44^l^°CD62L^+^) *Hdac1^f/f^Cd4Cre* CD8^+^ T cells displayed a normal proliferative response upon *in vitro* anti-CD3/anti-CD28 stimulation, produced similar amount of IL-2 and TNFα, while IFNγ production was slightly increased compared to *Hdac1^f/f^* CD8^+^ T cells upon activation. *Hdac1^f/f^* and *Hdac1^f/f^Cd4Cre* mice showed similar cytotoxic activity *in vivo*, suggesting that CTL function is not dependent on HDAC1. The infection with LCMV revealed reduced numbers of MHC class I/peptide-gp33 tetramer-positive (tet-gp33^+^) CD8^+^ T cells in the absence of HDAC1 correlating with impaired cytokine expression upon re-stimulation of splenocytes and liver cell suspensions in comparison to WT mice. Taken together, our data indicate that HDAC1 is required for the efficient generation of thymocytes and peripheral T cells, for proper CD8^+^ T cell homeostasis and for an efficient *in vivo* expansion and activation of CD8^+^ T cells in response to LCMV infection.

## Materials and Methods

### Ethics statement

All animal experiments were evaluated by the ethics committees of the Medical University of Vienna and approved by the Federal Ministry for Science and Research, Vienna, Austria (GZ:BMWF-66.009/0057-II/10b/2010 and GZ:BMWF-66.009/58-II/10b/2010). Animal husbandry and experimentation was performed under the national laws (Federal Ministry for Science and Research, Vienna, Austria) and ethics committees of the Medical University of Vienna and according to the guidelines of FELASA which match that of ARRIVE. Animals were euthanized by cervical dislocation. Experiments involving viral infections were performed in accordance with Swiss federal and cantonal guidelines under permission numbers SG11/05 and SG11/04 following review and approval by the Cantonal Veterinary Office (St. Gallen, Switzerland).

### Mice

*Hdac1^f/f^Cd4Cre* mice were previously described [16]. PLZF-luxoid (*Plzf^lu/lu^*) mice were kindly provided by A. Bendelac (University of Chicago). P14 were obtained from the European Mouse Mutant Archive, IL-15-null mice were obtained from Taconic. All mice were bred and maintained in the preclinical research facility of the Medical University of Vienna. Food and water was provided to the animals *ad libitum*.

### Antibodies used for flow cytometry

The following antibodies were used in this study: anti-CD3 (G4.18), anti-CD44 (IM7), anti-CD62L (MEL-14), anti-IL-2 (JES6-5H4) and anti-CD5 (53-7.3) were purchased from BD Bioscience. Anti-CD4 (RM4-5), anti-CD8 (53-6.7), anti-TCRβ (H57-597) and anti-B220 (RA3-6B2) were purchased from ebioscience. Anti-IFNγ (XMG1.2), anti-CD69 (H1.2F3) and anti-CD24 (M1/69) were purchased from Biolegend. Anti-HDAC1 (ABE260) and Anti-HDAC2 (3F3) were purchased from Millipore. Anti-rabbit IgG1 (H+L) was purchased from Invitrogen.

### Purification and in vitro activation of CD8^+^ T cells

Purification of CD8^+^ T cells was carried out as previously described [17]. In brief, spleens and lymph nodes of 6–10 week old mice were isolated and single cell suspensions were generated using a nylon cell strainer. After red blood cell lysis, cells were negatively depleted via MACS and naive (CD44^l^°CD62L^+^) CD8^+^ T cells were sorted on a FACSAria cell sorter (BD). Cells were seeded onto 48 well cell culture plates previously coated with anti-CD3 and anti-CD28 in the presence of 20 U/ml rhIL2 for 48 h, followed by splitting and resting for 2 days in presence of IL-2. For intracellular stainings, cells were restimulated with PMA/ionomycin for 4 h, followed by stainings performed as described [16], followed by flow cytometric analysis (LSRII, BD Biosciences). For ELISAs, cells were reactivated with platebound anti-CD3 for 16 hours. Supernatants were collected and ELISAs were performed using ebioscience “Ready-Set-Go” ELISA kits according to manufacturer’s instructions.

### CFSE labeling

Naive CD8^+^ T cells were isolated as described above, labeled with 1 µM CFSE (ebioscience), seeded onto cell culture plates and activated for 48 hours as described above.

### Intracellular HDAC1 and HDAC2 staining

Splenocytes were pre-incubated with Fc-block (BD Pharmingen) and stained with anti- CD8α, CD4 and TCRβ antibodies. For immunodetection of HDAC1 and HDAC2, cells were fixed and permeabilized using Foxp3 Fixation/Permeabilization Concentrate and Diluent (eBioscience) according to the manufacturer’s instruction. Subsequently, cells were blocked in 5% normal goat serum and then incubated with rabbit anti-mouse HDAC1 and mouse anti-mouse HDAC2 antibodies in permeabilization buffer (eBioscience) for 1 hour. Cells were washed with permeabilization buffer and incubated with Alexa Fluor 488-conjugated goat anti-rabbit IgG1 and biotinylated anti-mouse IgG1 antibodies, followed by a Streptavidin secondary staining.

### cDNA synthesis and quantitative real-time PCR

Cells were harvested with TRIZOL reagent (Invitrogen), and total RNA was isolated according the manufacturer’s instructions. RNA was reversely transcribed using the iScript cDNA synthesis kit (Bio-Rad). Quantitative real-time PCR (qRTPCR) analysis was performed with the SuperScript III qPCR MasterMix (Invitrogen) on the CFX 96 Real-Time PCR detection system (Bio-Rad).

### Primers used for quantitative real-time PCR

The following primers were used: *Eomes:* 5′- GCCTACCAAAACACGGATA, 5′-TCTGTTGGGGTGAGAGGAG; *Gzmb:* 5′-CCACTCTCGACCCTACATGG, 5′-GGCCCCCAAAGTGACATTTATT; *Hprt:* 5′-GATACAGGCCAGACTTTGTTG, 5′-GGTAGGCTGGCCTATAGGCT; *Prf1:* 5′-TTTCGCCTGGTACAAAAACC, 5′-CGTTCAGGCAGTCTCCTACC; *Runx3:* 5′-GTCAGCGTGCGACATGGCTTCCAACAG, 5′-AGCACGTCCATCGAGCGCACTTCGG; *Tbx21:* 5′-CAACAACCCCTTTGCCAAAG, 5′-TCCCCCAAGCAGTTGACAGT.

### Generation of bone marrow chimeric mice

BM transplantation was done as described [17]. Six to eight weeks after transplantation, the reconstituted mice were sacrificed and analyzed by flow cytometry (LSRII; BD Biosciences).

### In vivo cytotoxic T lymphocyte assays

CTL assays were performed as previously described [17]. In brief, *Hdac1^f/f^* and *Hdac1^f/f^Cd4Cre* mice were immunized by injecting 50 µl OVA peptide (SIINFEKL; 2 mg/ml in PBS)/adjuvant (CpG ODN1668; 1 mg/ml) or 50 µl PBS as a control into the footpads. After 4 days, target cells (3×10^7^) were i.v. injected into the tail vein of immunized mice. Target cells consisted of a 1∶1∶1 mixture of splenocytes that were either pulsed without peptide, with irrelevant peptide (VYDFFVWL, derived from murine tyrosine-related protein-2; Bachem) or with relevant OVA peptide (SIINFEKL). Splenocytes (1×10^7^ cells in 1 ml PBS) were pulsed with peptide (10 µg/ml) at 37° for 2 hours. Subsequently, “empty” splenocytes, or splenocytes pulsed with irrelevant or relevant peptide were labeled with 0.05, 0.5, and 5 µM CFSE respectively. Eight hours after target cell injection, immunized *Hdac1^f/f^* and *Hdac1^f/f^Cd4Cre* mice were sacrificed and the percentage of CFSE^low^, CSFE^med^ and CSFE^hi^ target cells in the draining lymph node was determined by flow cytometry. Specific killing was calculated as follows: % of specific killing = [1–(% of CFSE^hi^ cells/% of CFSE^low^ cells)]×100.

### LCMV infections: Cells and viruses

L929 cells were purchased from the European Collection of Cell Cultures. MC57 cells and lymphocyte choriomeningitis virus (LCMV) strain Armstrong were obtained from Dr. Zinkernagel (University of Zürich, Switzerland), the latter being propagated on L929 cells. Mice were infected intravenously with 200 pfu of LCMV Armstrong. LCMV virus titers were measured on MC57 cells using a focus-forming assay.

### LCMV infections: Antibodies, peptide MHC class I tetramers and *in vitro* restimulation

PE-conjugated tetramers against the LCMV gp33-41, and LCMV np396-404 were generated as previously described [18]. PE-conjugated tetramer for the LCMV gp34-41 was purchased from Sanquin (Amsterdam, The Netherlands).

For peptide-specific cytokine production, 10^6^ splenocytes were restimulated with gp33-41 peptide in the presence of Brefeldin A (5 µg/ml) for 5 hours at 37°C. Cells were stimulated with phorbolmyristateacetate (PMA, 50 ng/ml) and ionomycin (500 ng/ml; both purchased from Sigma) as positive control or left untreated as a negative control. For intracellular staining, restimulated cells were surface-stained and fixed with cytofix-cytoperm (BD Biosciences) for 20 min. Fixed cells were incubated at 4°C for 40 minutes with permeabilization buffer (2%FCS/0.5% Saponin/PBS) containing antibodies. Samples were analyzed by flow cytometry using a FACS Canto (Becton Dickinson). Gp33 peptide (KAVYNFATC) was purchased from Neosystem (Strasbourg, France).

### Statistical analysis

Unless otherwise stated the data are presented as mean ± SEM. GraphPad Prism software was used for data analysis and plotting. The P-values were calculated with two-tailed unpaired sample Student’s t-test where significance levels were set as following: *, P<0.05; **, P<0.01; ***, P<0.001.

## Results

### Homeostatic analysis of CD8^+^ T cells in *Hdac1^f/f^Cd4Cre* mice

**Figure 1:**
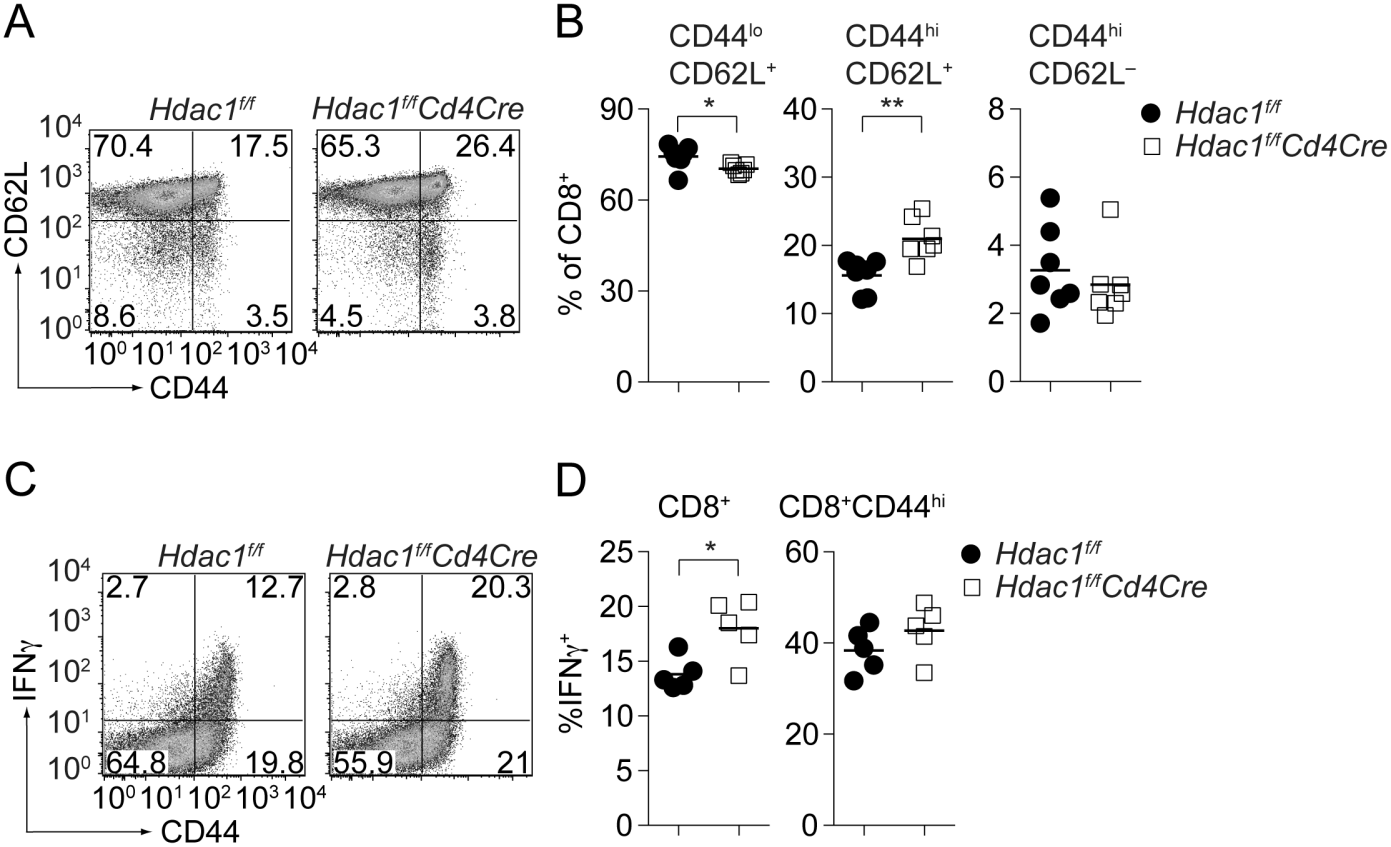
Increased CD44^hi^ CD8^+^ T cell subsets in *Hdac1^f/f^Cd4Cre* mice. (A) Plots depict CD44 and CD62L expression on splenic *Hdac1^f/f^* and *Hdac1^f/f^Cd4Cre* CD8^+^ T cells. Data shown are representative of 6 mice per genotype, analyzed in 4 independent experiments. (B) Diagrams showing the summary of the percentage of CD44^l^°CD62L^+^, CD44^hi^CD62L^+^ and CD44^hi^CD62L^–^ CD8^+^ T cells (mean ± SEM; n = 6). (C) *Hdac1^f/f^* and *Hdac1^f/f^Cd4Cre* splenocytes were stimulated *ex vivo* with PMA/ionomycin for 4 h and IFNγ production was analyzed by intracellular cytokine staining. Plots show IFNγ versus CD44 expression on gated CD8^+^ T cells. Data are representative of 5 mice analyzed in 3 independent experiments. (D) Summary of all PMA/ionomycin stimulation experiments as described in C. The left diagram depicts the percentage of IFNγ-producing CD8^+^ T cells, while the diagram to the right indicates the percentage of IFNγ-producing cells within the CD44^hi^CD8^+^ T cell population. Mean ± SEM is shown (n = 5). (C, D) Numbers in the dot plot indicate the percentage of cells in the respective quadrants. (B, D) Statistical analysis was performed by using a two-tailed non-paired Student’s t test. The P-values were defined as following: *, P<0.05; **, P<0.01; ***, P<0.001.

We previously reported that there are no major alterations during T cell development or in the composition of peripheral T cell subsets in *Hdac1^f/f^Cd4Cre* mice [16]. A careful re-analysis of peripheral T cell subsets revealed a slightly increased population of CD8^+^ T cells with a CD44^hi^CD62L^+^ memory-like phenotype (Fig. 1A and 1B and Fig. S1A). To test whether the increase in CD44^hi^CD62L^+^ CD8^+^ T cells correlated also with an increase in IFNγ production of CD44^hi^ HDAC1-null CD8^+^ T cells, splenocytes were isolated and stimulated *ex vivo* with PMA/ionomycin. While the percentage of IFNγ-producing cells within the total peripheral CD8^+^ T cell population was significantly enhanced in the absence of HDAC1 (Fig. 1C and 1D), there was no difference in the percentage IFNγ-producing cells within the CD44^hi^ CD8^+^ T cell subset (Fig. 1D). This indicates that CD44^hi^ effector CD8^+^ T cells are enhanced in *Hdac1^f/f^Cd4Cre* mice, while IFNγ production in CD8 effector T cells is not affected by the absence of HDAC1.

Next, we wanted to determine whether peripheral CD44^hi^ CD8^+^ T cells are enhanced due to T cell developmental defects that can be detected already at late stages during T cell maturation. There was no increase in CD44^hi^ cells within the mature CD8SP subsets and CD8SP cells showed a normal expression pattern of CD5, CD24 and CD69 (Fig. S1B. Moreover, PMA/ionomycin stimulation did not indicate enhanced IFNγ production (Fig. S1C), suggesting that cytokine producing CD44^hi^ subsets are not enhanced within the CD8SP population.

### T cell developmental defects in *Hdac1^f/f^LckCre* mice

**Figure 2:**
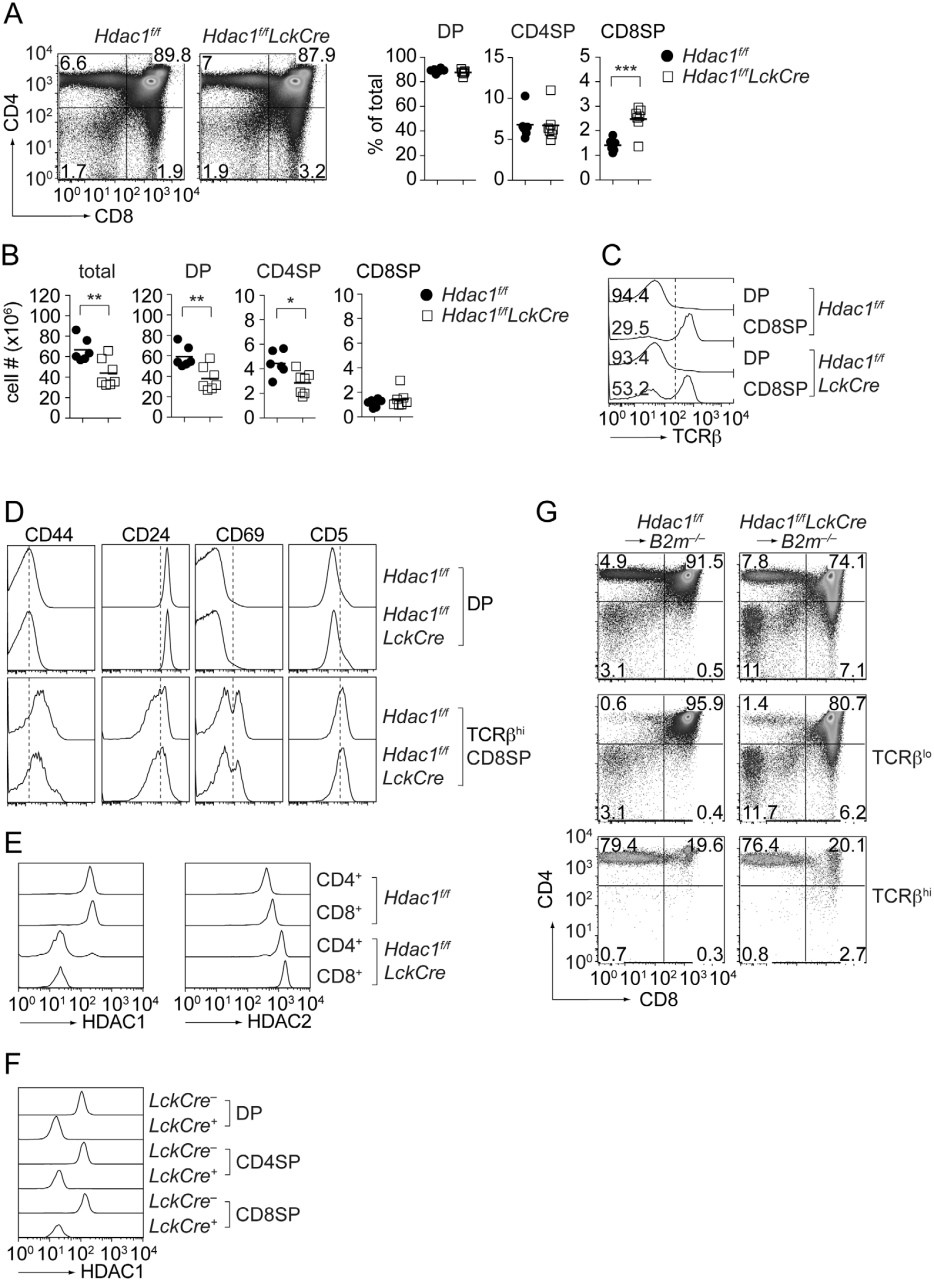
LckCre-mediated deletion of HDAC1 leads to T cell developmental defects. (A) CD4/CD8 plots from *Hdac1^f/f^* and *Hdac1^f/f^LckCre* thymocytes. The percentage of DP, CD4SP and CD8SP thymocyte subsets of all mice analyzed is shown in the diagrams at the right (mean ± SEM; n = 6 for *Hdac1^f/f^* and n = 7 for *Hdac1^f/f^LckCre* mice, analyzed in 4 independent experiments). (B) Thymocytes (total) and thymocyte subset cell numbers of *Hdac1^f/f^* (n = 6) and *Hdac1^f/f^LckCre* (n = 7) mice (mean ± SEM is shown). (C) Histograms depict TCRβ expression levels on *Hdac1^f/f^* and *Hdac1^f/f^LckCre* DP and CD8SP thymocytes. The numbers indicate the percentage of TCRβ^lo^ cells. Data are representative of 5 mice analyzed in 3 independent experiments. Numbers indicate the fraction of TCRβ^lo^ cells within the indicated thymocyte subsets. (D) CD44, CD24, CD69 and CD5 expression levels on *Hdac1^f/f^* and *Hdac1^f/f^LckCre* DP and CD8SP thymocytes. Data are representative of 5 mice analyzed in 3 independent experiments. (E) Intracellular HDAC1 and HDAC2 expression levels in splenic *Hdac1^f/f^* and *Hdac1^f/f^LckCre* T cells. Data are representative of two mice. (F) Intracellular HDAC1 expression levels in thymic subsets isolated from *Hdac1^f/f^* and *Hdac1^f/f^LckCre* mice. Data are representative of two mice. (G) *Hdac1^f/f^* and *Hdac1^f/f^LckCre* BM cells were transplanted into irradiated MHC class I-deficient (*B2 m^−/−^*) mice. Eight weeks after reconstitution, BM chimeric *B2 m^−/−^* mice were analyzed for the expression of CD4 and CD8 on total thymocytes (upper panel), and on gated TCRβ^lo^ (middle panel) and TCRβ^hi^ (lower panel) cells. Data are representative of 4 BM chimeric *B2 m^−/−^* mice generated in 2 independent experiments. (A, G) Numbers in the dot plot indicate the percentage of cells in the respective quadrants. (A, B) Statistical analysis was performed by using a two-tailed non-paired Student’s t test. The P-values were defined as following: *, P<0.05; **, P<0.01; ***, P<0.001.

We previously showed that *Cd4Cre* mice had efficiently deleted *Hdac1* alleles at the DP stage [16]. However, probably due to a slow turnover, HDAC1 protein is still detectable in DP and CD4SP thymocytes and was only fully absent in peripheral CD4^+^ and CD8^+^ T cells [16]. We confirmed these findings using intracellular HDAC1 staining approaches and detected some residual HDAC1 protein also in CD8SP thymocytes (Fig. S1D). This residual expression of HDAC1 protein might mask potential developmental defects within the CD8SP population in *Hdac1^f/f^Cd4Cre* mice. Therefore, *Hdac1^f/^* ^f^ mice were crossed with the *LckCre* deleter strain and thymocyte development was assessed. While the relative percentage of DP cells and CD4SP thymocytes was similar between *Hdac1^f/f^* and *Hdac1^f/f^LckCre* mice (Fig. 2A), the CD8SP subset was increased in *Hdac1^f/f^LckCre* thymii (Fig. 2A). Of note, *Hdac1^f/f^LckCre* mice had reduced total thymocyte numbers accompanied with a reduction of DP and CD4SP cells, while CD8SP numbers were not reduced in *Hdac1^f/f^LckCre* mice (Fig. 2B). The assessment of CD3/TCRβ expression revealed an increased fraction of CD3/TCRβ^lo^ cells within the CD8SP population in *Hdac1^f/f^LckCre* mice (Fig. 2C). The *Hdac1^f/f^LckCre* CD3/TCRβ^hi^ CD8SP population had a similar expression pattern of CD69, CD5 and CD24, and there were also similar percentages of CD44^hi^ cells within the CD8SP population (Fig. 2D). Intracellular staining revealed that HDAC1 protein was deleted in peripheral CD4^+^ and CD8^+^ T cells (Fig. 2E) and this was accompanied with a compensatory up-regulation of HDAC2 (Fig. 2E). During T cell development, HDAC1 was efficiently deleted already in DP thymocytes (Fig. 2F). To test whether the enhanced fraction of CD3/TCRβ^l^°CD8^+^ cells is due to an increase in immature CD8SP thymocytes (ISP) or due to a failure of MHC class I-signaled DP thymocytes to differentiate into mature CD8SP thymocytes, we generated BM chimeric MHC class I-null mice. *Hdac1^f/f^* or *Hdac1^f/f^LckCre* BM cells were transferred into irradiated MHC class I-null recipient mice and T cell development was analyzed 8 weeks after the transfer. HDAC1-null BM chimeric MHC class I-null mice developed in addition to DP and CD4SP cells also a significantly enlarged fraction of CD8SP thymocytes (Fig. 2G). This indicates that the enhanced fraction of CD8SP cells in *Hdac1^f/f^LckCre* mice is due to an increase in immature CD8SP thymocytes.

**Figure 3:**
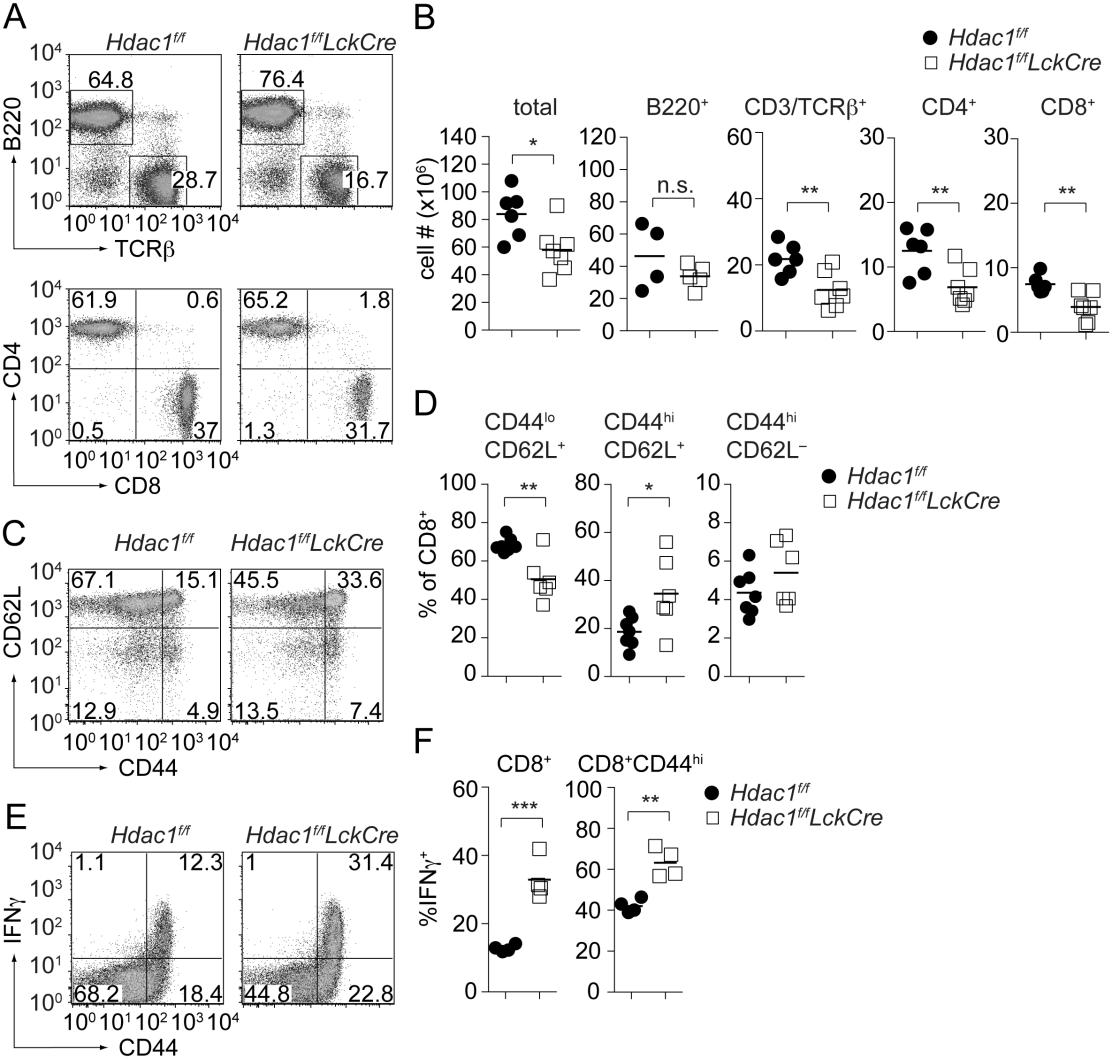
Reduced peripheral T cell numbers but enhanced CD44^hi^ CD8^+^ T cell populations in *Hdac1^f/f^LckCre* mice. (A) TCRβ and B220 (upper panels), and CD4 and CD8 (lower panels, gated on TCRβ^+^ cells) expression on *Hdac1^f/f^* and *Hdac1^f/f^LckCre* splenocytes. Data are representative for 6 (*Hdac1^f/f^*) and 7 (*Hdac1^f/f^LckCre*) mice analyzed in 4 independent experiments. (B) Cell numbers of the indicated *Hdac1^f/f^* (n = 6) and *Hdac1^f/f^LckCre* (n = 7) splenic immune cell subsets (mean ± SEM is shown; for B220^+^ subsets: n = 4 and 5 mice analyzed in 3 independent experiments, respectively). (C) CD44/CD62L expression on peripheral *Hdac1^f/f^* and *Hdac1^f/f^Cd4Cre* CD8^+^ T cells. Numbers in the dot plot indicate the percentage of cells in the respective quadrants. (D) The summary of the percentage of CD44^l^°CD62L^+^, CD44^hi^CD62L^+^ and CD44^hi^CD62L^–^ *Hdac1^f/f^* (n = 6) and *Hdac1^f/f^LckCre* (n = 7) and CD8^+^ T cells is shown (mean ± SEM). Mice were analyzed in 4 independent experiments (E) *Hdac1^f/f^* and *Hdac1^f/f^LckCre* splenocytes were stimulated *ex vivo* with PMA/ionomycin for 4 h and IFNγ production was analyzed by intracellular cytokine staining. Plots show IFNγ versus CD44 expression on gated CD8^+^ T cells. Data are representative of 4 mice analyzed in 2 independent experiments (F) Summary of all PMA/ionomycin stimulation experiments as described in E. The left diagram depicts the percentage of IFNγ-producing CD8^+^ T cells, while the diagram to the right indicates the percentage of IFNγ-producing cells within the CD44^hi^CD8^+^ T cell population (mean ± SEM; n = 4). (A, C, E) Numbers in the plots indicate the percentage of cells in the respective regions or quadrants. (B, D, F) Statistical analysis was performed by using a two-tailed non-paired Student’s t test. The P-values were defined as following: *, P<0.05; **, P<0.01; ***, P<0.001.

In agreement with the reduced numbers of thymocytes, the percentage and numbers of peripheral T cells were reduced in *Hdac1^f/f^LckCre* mice, however the CD4/CD8 T cell ratio was normal (Fig. 3A and 3B). Even though CD44^hi^ cells were not enhanced within the thymic CD3^hi^CD8SP population (Fig. 2D), an increase in peripheral CD44^hi^CD62L^+^ CD8^+^ T cells was observed in *Hdac1^f/f^LckCre* mice (Fig. 3C and 3D), similar to the observations in *Hdac1^f/f^Cd4Cre* mice. The CD44^hi^CD62L^+^ CD8^+^ T cell fraction in *Hdac1^f/f^LckCre* mice (approx. 35%, Fig. 3D) was even more enhanced compared to *Hdac1^f/f^Cd4Cre* mice (approx. 21%, Fig. 1A), indicating that the *LckCre*-mediated earlier loss of HDAC1 in comparison to *Cd4Cre*-mediated deletion has a stronger effect on peripheral T cell homeostasis. *Ex vivo* PMA/ionomycin stimulations of peripheral T cells revealed that the percentage of IFNγ-producing CD44^hi^ cells was increased in *Hdac1^f/f^LckCre* mice (Fig. 3E and 3F), indicating altered peripheral CD8^+^ T cell subset composition. Peripheral CD4^+^ T cell homeostasis was also altered in *Hdac1^f/f^LckCre* mice, since there was a mild reduction of naïve CD44^l^°CD62L^+^ T cells within the HDAC1/2-null CD4^+^ T cell population (Fig. S2A and S2B).

### HDAC1-null CD8^+^ T cells display enhanced cytokine expression

**Figure 4:**
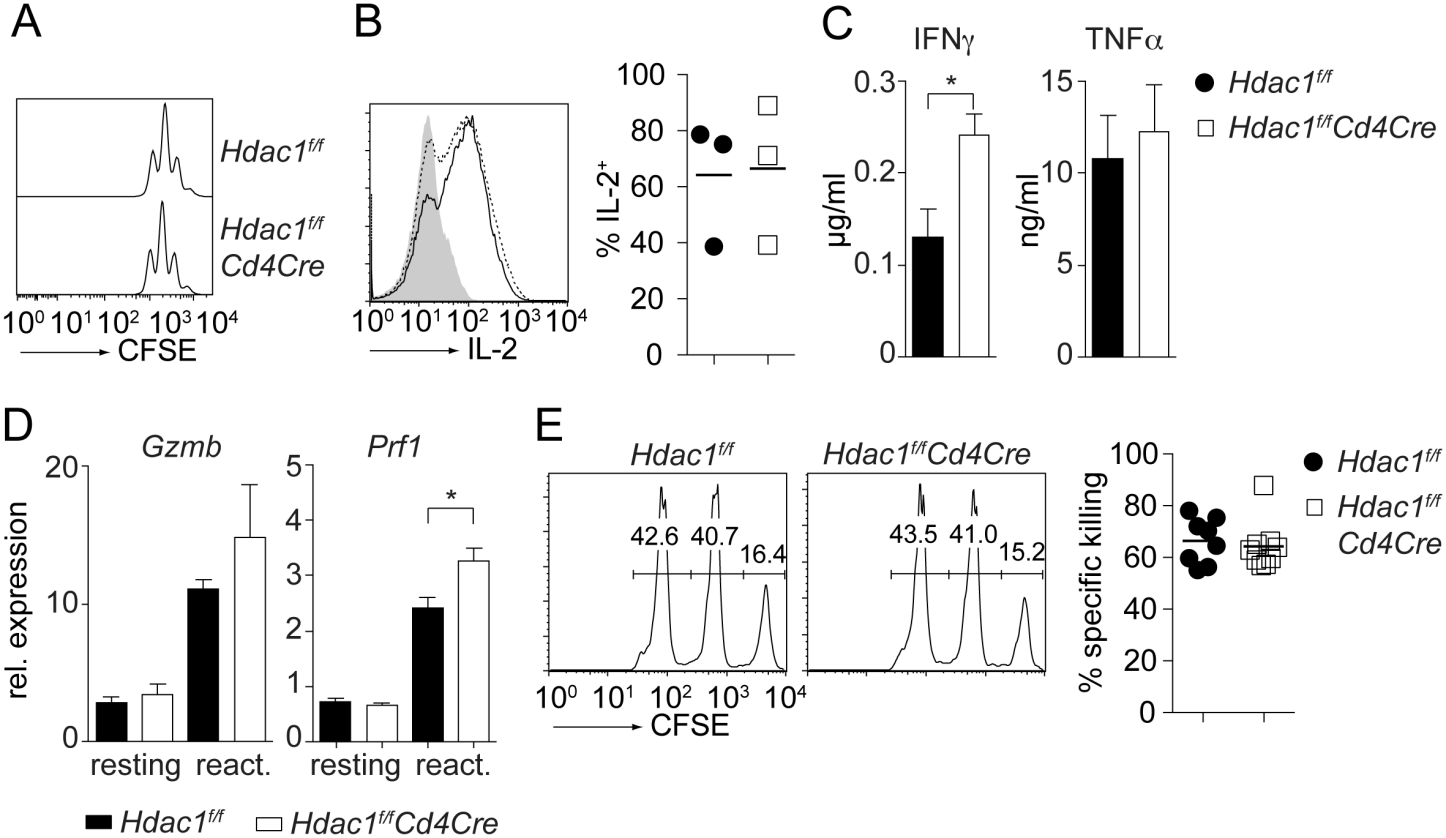
CTL effector functions in the absence of HDAC1. (A) Naïve *Hdac1^f/f^* and *Hdac1^f/f^Cd4Cre* CD8^+^ T cells were labeled with CFSE and stimulated with anti-CD3/anti-CD28. Histograms depict CFSE intensity after 48 h of stimulation. Data are representative of 5 independent experiments. (B) Naïve cells were stimulated with anti-CD3/anti-CD28 for 48 hours. Cells were split 1∶2 on day 2, cultured for 2 additional days and stimulated with PMA/ionomycin for 4 hours. Histogram depicts intracellular IL-2 expression and the percentage of IL-2-expressing cells for all experiments is shown at the right (mean ± SEM; n = 3, performed in 3 independent experiments). (C) Naïve cells were stimulated with anti-CD3/anti-CD28. Cells were split 1∶2 on day 2, cultured for 2 additional days and re-stimulated with anti-CD3 overnight. IFNγ and TNFα levels in the supernatant were determined by ELISA (mean ± SEM; n = 3, performed in 3 independent experiments). (D) Naïve *Hdac1^f/f^* and *Hdac1^f/f^Cd4Cre* CD8^+^ T cells were activated as described in C. The expression of *Gzmb* and *Prf1* was assessed by qRTPCR before (“resting”) and after (“react.”) overnight restimulation with anti-CD3 and normalized to *Hprt1* expression (mean ± SEM; n = 3, performed in 3 independent experiments). (E) *Hdac1^f/f^* and *Hdac1^f/f^Cd4Cre* mice were immunized with OVA peptide (SIINFEKL) plus adjuvant. Subsequently, target cells were intravenously injected 4 days post-immunization. Target cells consisted of a 1∶1∶1 mixture of CFSE-labeled splenocytes that were either pulsed with OVA peptide (CFSE^hi^), with irrelevant peptide (CFSE^med^) or without peptide (CFSE^low^). Eight hours after target-cell injection, the percentage of CFSE^low^, CSFE^med^ and CSFE^hi^ target cells in the draining lymph node of *Hdac1^f/f^* and *Hdac1^f/f^Cd4Cre* mice was determined. Numbers in the histogram show the percentage of cells within the indicated regions. The diagram at the right indicates the percentage of specific lysis (as defined in Materials and Methods) and shows the summary of all *in vivo* CTL experiments performed (n = 8 for *Hdac1^f/f^*; n = 9 for *Hdac1^f/f^Cd4Cre* mice, analyzed in 2 independent experiments). (B, C, D, E) Statistical analysis was performed using a two-tailed non-paired Student’s t test. The P-values were defined as following: *, P<0.05; **, P<0.01; ***, P<0.001.

HDAC1 is a crucial modulator of Th2 cytokine expression in CD4^+^ T cells [16]. To study whether HDAC1 controls the extent of cytokine expression in CD8^+^ T cells, we sorted naïve (CD44^l^°CD62L^+^) wild-type and HDAC1-null CD8^+^ T cells and activated them with anti-CD3/anti-CD28. In order to exclude any alterations in CD8^+^ T cells due to potential developmental defects upon early *LckCre*-mediated loss of HDAC1, CD8^+^ T cells were isolated from *Hdac1^f/f^* and *Hdac1^f/f^Cd4Cre* mice. Wild-type and HDAC1-null CD8^+^ T cells showed a similar proliferation rate upon activation (Fig. 4A) and produced similar levels of IL-2 (Fig. 4B) and TNFα (Fig. 4C), while IFNγ production HDAC1-null CD8^+^ T cells was slightly enhanced (Fig. 4C).

Next, we analyzed whether HDAC1 regulates CTL effector function. Runx3, T-bet and Eomesodermin are key transcriptional regulators of CTL differentiation and function [19]–[24]. *Runx3d* and *Tbx21* (encoding for T-bet) were expressed at similar levels in HDAC1-null CD8^+^ T cells in comparison to wild-type CD8^+^ T cells, while *Eomes* was slightly up-regulated in the absence of HDAC1 (Fig. S3). Upon activation, HDAC1-null CD8^+^ T cells expressed wild-type levels of *GzmB* (encoding for Granzyme B), while *Prf1* (encoding for Perforin) expression was slightly enhanced in the absence of HDAC1 (Fig. 4D). To test whether the up-regulation of Perforin correlated with increased CTL activity in the absence of HDAC1, *in vivo* CTL assays were performed. This revealed that *Hdac1^f/f^* and *Hdac1^f/f^Cd4Cre* mice had a similar CTL activity *in vivo* (Fig. 4E). Together, this indicates that HDAC1 has only a minor role in the *in vitro* differentiation of CD8 effector T cells.

### Loss of HDAC1 leads to reduced proliferation and expansion of gp33-specific CD8^+^ T cells in response to LCMV infection

**Figure 5:**
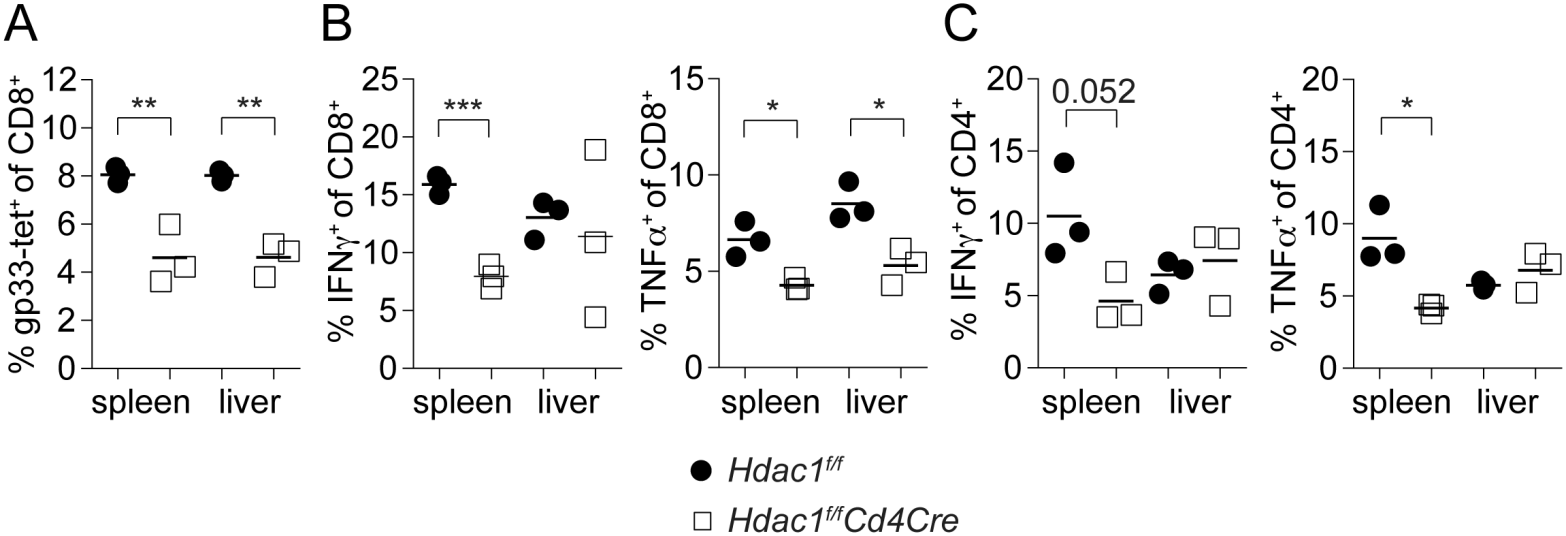
*Hdac1^f/f^Cd4Cre* mice show impaired CD8^+^ T cell responses upon LCMV infection. (A) *Hdac1^f/f^* and *Hdac1^f/f^Cd4Cre* mice were infected i.v. with 200 pfu LCMV (Armstrong). On day 8, spleen and liver were isolated and cells were analyzed. The percentage of viral-specific CD8^+^ T cells was determined using MHC class I tetramers specific for the viral peptides gp33 (tet-gp33). Diagram shows the percentage of tet-gp33^+^ CD8^+^ T cell populations isolated from spleen and liver of *Hdac1^f/f^* and *Hdac1^f/f^Cd4Cre* mice. Mean ± SD is shown (n = 3, analyzed in 1 experiment). (B) Mice were infected as described in A. On day 8, spleens and livers were isolated and cell suspensions were re-stimulated with gp33 peptide for 5 hours. IFNγ and TNFα expression was determined by intracellular cytokine staining. The percentage of INFγ^+^ or of TNFα^+^ producing CD8^+^ T cells is shown (mean ± SEM; n = 3, analyzed in 1 experiment). (C) Mice were infected as described in A. On day 8, spleen and liver were isolated and cell suspensions were re-stimulated with the MHC class II-specific peptide gp64–80 (P13) for 5 hours. IFNγ and TNFα expression was determined by intracellular cytokine staining. The percentage of INFγ^+^ and TNFα^+^ producing CD4^+^ T cells is shown (mean ± SEM; n = 3, analyzed in 1 experiment). (A–C) Statistical analysis was performed using a two-tailed non-paired Student’s t test. The P-values were defined as following: *, P<0.05; **, P<0.01; ***, P<0.001.

To study the *in vivo* CD8^+^ T cell response in the absence of HDAC1 in response to a pathogen, *Hdac1^f/f^* and *Hdac1^f/f^Cd4Cre* were i.v. infected with LCMV (Armstrong). On day 8, the spleen and liver of infected mice were isolated and the percentage of virus-specific CD8^+^ T cells was determined using MHC class I tetramers specific for the viral peptides GP33 (tet-gp33). This revealed reduced numbers of tet-gp33^+^ CD8^+^ T cells in the liver and spleen in the absence of HDAC1 (Fig. 5A), which correlated with reduced percentages of INFγ^+^ and TNFα^+^ splenic and reduced TNFα^+^ liver HDAC1-null CD8^+^ T cells in comparison to wild-type CD8^+^ T cells upon re-stimulation of splenocytes and liver cell suspensions with gp33 (Fig. 5B). A similar tendency of reduced tet-gp33^+^ CD8^+^ T cells and reduced IFNγ production was also seen on day 6 post-infection (Fig. S4). Interestingly, splenic HDAC1-null CD4^+^ T cells responses were also reduced upon re-stimulation with the MHC class II-specific peptide gp64-80 (P13) in comparison to wild-type CD4^+^ T cells, indicated by reduced percentages of INFγ^+^ and TNFα^+^ CD4^+^ T cells in the spleen but not liver of LCMV-infected *Hdac1^f/f^Cd4Cre* mice (Fig. 5C).

**Figure 6:**
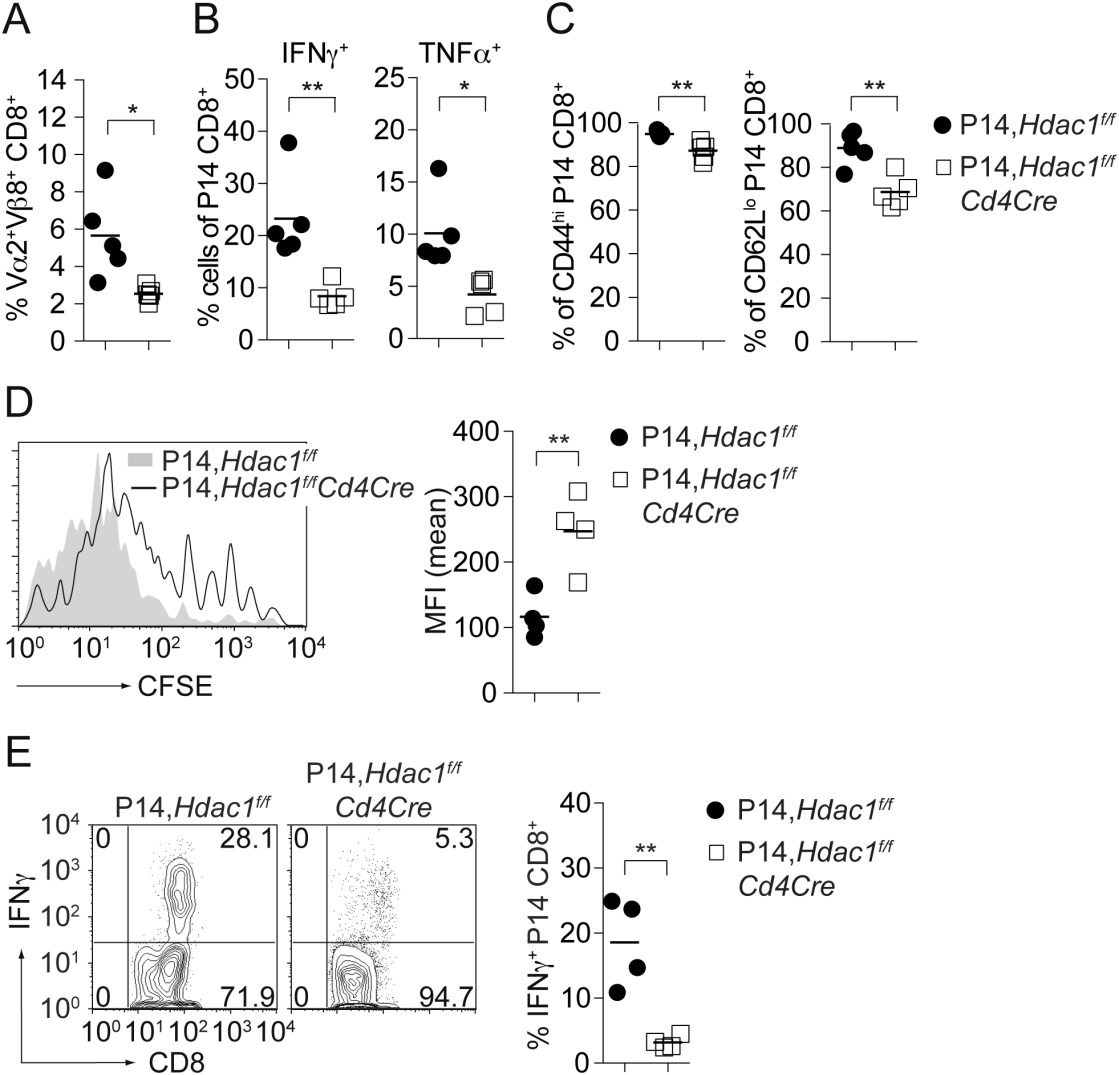
Adoptively transferred HDAC1-null transgenic P14 CD8^+^ T cells display impaired expansion and activation. (A) *Hdac1^f/f^* and *Hdac1^f/f^Cd4Cre* P14 CD8^+^ T cells (10^4^ cells) were adoptively transferred into C57BL/6 mice. The next day, recipient mice were infected i.v. with 200 pfu LCMV Armstrong. The percentage of Vα2/Vβ8-expressing transgenic P14 CD8^+^ T cells on day 7 is shown (mean ± SEM; n = 5, analyzed in 1 experiment). (B) Mice were prepared and infected as described in A and the anti-LCMV-specific CD8^+^ T cell response was determined upon *in vitro* re-stimulation with the gp33 peptide. Diagrams show the percentage of INFγ^+^ and TNFα^+^ expressing CD45.2^+^ CD8^+^ T cells upon re-stimulation (mean ± SEM; n = 5, analyzed in 1 experiment). (C) Diagram showing the percentage of CD44^hi^ (left) and CD62L^lo^ (right) transgenic P14 CD8^+^ T cells. Data show mean ± SEM (n = 5, analyzed in 1 experiment). (D) CFSE labeled *Hdac1^f/f^* and *Hdac1^f/f^Cd4Cre* P14 CD8^+^ T cells (10^6^ cells; CD45.2^+^) were adoptively transferred into CD45.1^+^ C57BL/6 mice. Recipient mice were infected with 200 pfu LCMV Armstrong. Histogram shows CFSE intensity in CD45.2^+^ *Hdac1^f/f^* and *Hdac1^f/f^Cd4Cre* P14 CD8^+^ T cells on day 5 after infection. The diagram at the right indicates the mean fluorescence expression (MFI) of CFSE (mean ± SD; n = 4, analyzed in 1 experiment). (E) Mice were prepared and infected as described in A (lower panel). Splenic cells were isolated on day 5 and restimulated with gp33 peptide. Contour plots to the left show intracellular IFNγ versus CD8 expression. Diagram to the right shows the summary of all experiments performed (mean ± SEM; n = 4, analyzed in 1 experiment). The numbers indicate the percentage of cells in the respective quadrants. (A–E) Statistical analysis was performed using a two-tailed non-paired Student’s t test. The P-values were defined as following: *, P<0.05; **, P<0.01; ***, P<0.001.

To determine whether the altered CD8^+^ T cell responses in *Hdac1^f/f^Cd4Cre* mice are due to CD8^+^ T cell-intrinsic alteration, we crossed *Hdac1^f/f^Cd4Cre* mice with P14 TCR transgenic mice that express TCRs (V_β_8 and V_α_2) specific for gp33 in the context of MHC class I [25]. *Hdac1^f/f^Cd4Cre*,P14 T cells did not display any obvious developmental alterations and the fraction of V_α_2^+^ T cells was similar between *Hdac1^f/f^* and *Hdac1^f/f^Cd4Cre* P14 tg mice (data not shown). *Hdac1^f/f^* and *Hdac1^f/f^Cd4Cre*,P14 CD8^+^ T cells were adoptively transferred into C57BL/6 mice. The next day, recipient mice were infected with 200 pfu LCMV Armstrong and on day 7 the anti-LCMV-specific CD8^+^ T cell response was determined upon *ex vivo* re-stimulation with gp33 peptide. In line with the observations in LCMV-infected *Hdac1^f/f^Cd4Cre* mice, HDAC1-null P14 CD8^+^ T cells displayed a significant reduction of V_β_8^+^/V_α_2^+^ CD8^+^ T cells (Fig. 6A), suggesting impaired expansion of HDAC1-null CD8^+^ T cells. Moreover, HDAC1-null P14 CD8^+^ T cells produced less IFNγ and TNFα in comparison to transferred HDAC1-sufficient P14 CD8^+^ T cells (Fig. 6B). In addition, the HDAC1-null P14 CD8^+^ T cell population displayed lower percentages of CD44^hi^ and CD62L^lo^ cells, again indicating reduced activation (Fig. 6C). To better evaluate the proliferation/expansion properties of HDAC1-null CD8^+^ T cells upon *in vivo* infection, CFSE-labeled *Hdac1^f/f^* and *Hdac1^f/f^Cd4Cre* P14 CD8^+^ T cells (CD45.2^+^) were adoptively transferred into C57BL/6 (CD45.1^+^) mice. The next day, recipient mice were infected with LCMV and on day 5 the proliferation history of transferred P14 CD8^+^ T cells was determined. This revealed reduced proliferation of HDAC1-null P14 CD8^+^ T cells (Fig. 6D) as well as severely reduced IFNγ production upon gp33 re-stimulation (Fig. 6E). Collectively, these data indicate that HDAC1 is required for an efficient *in vivo* expansion and activation of CD8^+^ T cells in response to LCMV infection.

## Discussion

In this study we investigated how loss of HDAC1 affects the differentiation of effector CD8^+^ T cells. Our data indicate that HDAC1 is required for the efficient generation of thymocytes and peripheral T cells, for proper CD8^+^ T cell homeostasis and for an efficient *in vivo* expansion and activation of CD8^+^ T cells in response to LCMV infection. Thus, our study reveals an immune regulatory function of HDAC1 in CD8 lineage T cells.

We observed an increase in CD8^+^ T cells with a CD44^hi^CD62L^+^ effector/memory phenotype in the absence of HDAC1. Although HDAC1 was efficiently deleted in peripheral CD4^+^ and CD8^+^ T cells using the *Cd4Cre* delete strain [16], HDAC1 protein was still detectable in CD4SP thymocytes [16] as well as in CD8SP cells, most likely due to a slow turnover. The slowly disappearing HDAC1 protein expression in *Hdac1^f/f^Cd4Cre* mice makes it difficult to dissect developmental alterations during late stages of T cell development from peripheral defects. However, HDAC1 protein was absent in CD4SP and CD8SP thymocytes upon *LckCre*-mediated early-stage deletion. This correlated with an enhanced fraction of CD44^hi^CD62L^+^ CD8^+^ T cells in comparison to *Hdac1^f/f^Cd4Cre* mice, indicating that efficient loss of HDAC1 in CD8SP thymocytes leads to developmental alterations resulting in enhanced CD44^hi^ subsets. However, TCRβ^hi^ HDAC1-null CD8SP thymocytes displayed a similar expression pattern of maturation markers, suggesting that positive selection is not severely affected in the absence of HDAC1.

The observation that CD44^hi^ cells are enhanced in *Hdac1^f/f^Cd4Cre* and *Hdac1^f/f^LckCre* mice raises the question about the cellular nature of HDAC1-null CD44^hi^ T cells. CD44^hi^ T cells represent a heterogeneous population consisting of effector/memory T cells known to be dependent on IL-15 [26], [27]. In addition, CD44^hi^ subsets contain also innate-like T cells that are able to rapidly produce cytokines in response to activation, and the development of these cells is dependent on PLZF [28]. Preliminary results with *Hdac1^f/f^LckCre* mice either carrying the luxoid mutation of PLZF (*Plzf ^lu/lu^*), which leads to an inactivated form of PLZF [29] or with IL-15-null mice [27] indicated that there was a similar relative reduction in the percentage of CD44^hi^ cells in the absence of either PLZF or of IL-15 between *Hdac1^f/f^* and *Hdac1^f/f^LckCre* mice (Fig. S5). Thus, it is unlikely that the enhanced CD44^hi^ population in *Hdac1^f/f^LckCre* mice is due to the preferential development/expansion of a PLZF- or an IL-15-dependent CD8^+^ T cell subset.

*Hdac1^f/f^LckCre* mice had reduced numbers of total thymocytes as well as peripheral T cells. This is in contrast to a previous study, which reported similar numbers of thymocytes in *Hdac1^f/f^LckCre* mice in comparison to wild-type mice [6]. However, the other study did not investigate T cell development in much detail and peripheral T cell subsets were not analyzed. Finally, we observed that the population of TCRβ^lo^ immature CD8 single-positive thymocytes was increased among *Hdac1^f/f^LckCre* thymocytes. Together, our data strongly indicate that *LckCre*-mediated deletion of HDAC1 affects T cell development and peripheral T cell homeostasis.

Another finding of our study is the observation that *in vitro* CTL differentiation and *in vivo* CTL activity is largely unaffected in the absence of HDAC1. We observed a compensatory up-regulation of HDAC2 in CD8^+^ T cells similar to reports for other cell lineages such as CD4^+^ T cells [16], B cells [30], during erythrocyte-megakaryocyte differentiation [31] and in non-hematopoietic cells [32]–[35]. Thus it is likely that this closely related class I HDAC family member partially compensates for loss of HDAC1 and thus masks potential functional consequences of HDAC1 deletion. However, HDAC1 was essential for a proper anti-LCMV response *in vivo*. LCMV-infected *Hdac1^f/f^Cd4Cre* mice displayed reduced numbers of virus-specific tet-gp33^+^ CD8^+^ T cells as well as reduced cytokine production upon re-stimulation, although the number of virus-specific tet-np396^+^ T cells and the clearance of LCMV virus was not altered (data not shown). This suggests that HDAC1-null CD8^+^ T cells might react differentially to the signaling strength/quality of different viral-peptide–MHC complexes. The reduced anti-gp33^+^ CD8^+^ T cell response was not due to impaired CD4^+^ T cell responses in the absence of HDAC1, since *Hdac1^f/f^Cd4Cre* P14 TCR transgenic CD8^+^ T cells that were adoptively transferred into wild-type mice displayed a similar reduction in the percentage of anti-viral gp33-specific CD8^+^ T cells as well as cytokine production in comparison to transferred wild-type P14 CD8^+^ T cells. Moreover, CFSE labeling experiments combined with adoptive transfer approaches indicated reduced proliferation, suggesting that virus-specific tet-gp33^+^ T cells are reduced due to impaired expansion rather than increased apoptosis. Collectively, these data indicate that HDAC1 is required for an efficient *in vivo* expansion and activation of CD8^+^ T cells in response to LCMV infection.

## Supporting Information

Figure S1 **Characterization of T cell and thymocyte subsets in** ***Hdac1^f/f^Cd4Cre*** **mice.** (A) Diagrams showing cell numbers of *Hdac1^f/f^* and *Hdac1^f/f^Cd4Cre* splenic subpopulations (mean ± SEM; n = 6, performed in 4 independent experiments). Cell numbers for total splenocytes and CD3^+^ T cells are in agreement with previously published data (16). (B) CD44, CD24, CD69 and CD5 expression levels on *Hdac1^f/f^* and *Hdac1^f/f^Cd4Cre* CD8SP thymocytes (n = 4, performed in 2 independent experiments). (C) Thymocytes isolated from *Hdac1^f/f^* and *Hdac1^f/f^Cd4Cre* mice were stimulated *ex vivo* with PMA/ionomycin for 4 h and IFNγ production was analyzed by intracellular cytokine staining. Mean ± SEM is shown (n = 4, performed in 2 independent experiments). Cells were gated on CD8SP thymocytes. (D) Intracellular HDAC1 levels in *Hdac1^f/f^* (solid grey) and *Hdac1^f/f^Cd4Cre* (black line) CD8SP thymocytes. Data shown are representative of 2 independent experiments. (A, C) Statistical analysis was performed using a two-tailed non-paired Student’s t test. The P-values were defined as following: *, P<0.05; **, P<0.01; ***, P<0.001; n.s., not significant.
(TIF)

Figure S2 **The distribution of naïve and memory CD4^+^ T cells in** ***Hdac1^f/f^LckCre*** **mice.** (A) CD44/CD62L expression on peripheral *Hdac1^f/f^* and *Hdac1^f/f^Cd4LckCre* CD4^+^ T cells. Numbers in the plots indicate the percentage of cells in the respective quadrants. (B) The summary of the percentage of CD44^l^°CD62L^+^, CD44^hi^CD62L^+^ and CD44^hi^CD62L^–^ *Hdac1^f/f^* (n = 6) and *Hdac1^f/f^LckCre* (n = 7) CD4^+^ T cells is shown (mean ± SEM; performed in 4 independent experiments). Statistical analysis was performed using a two-tailed and non-paired Student’s t test. The P-values were defined as following: *, P<0.05; **, P<0.01; ***, P<0.001; n.s., not significant.
(TIF)

Figure S3 **Expression of key transcription factors in** ***Hdac1^f/f^Cd4Cre*** **CD8^+^ T cells.** Naïve *Hdac1^f/f^* and *Hdac1^f/f^Cd4Cre* CD8^+^ T cells were stimulated with anti-CD3/anti-CD28 for 48 hours. Cells were split 1∶2 on day 2, cultured for 2 additional days and re-stimulated with anti-CD3 overnight. The expression of *Tbx21*, *Runx3d* and *Eomes* was assessed by qRTPCR before (“resting”) and after (“react.”) overnight restimulation with anti-CD3. Expression was normalized to *Hprt1* expression (mean ± SEM; n = 3; performed in 3 independent experiments). Statistical analysis was performed using a two-tailed and non-paired Student’s t test. The P-values were defined as following: *, P<0.05.
(TIF)

Figure S4 ***Hdac1^f/f^Cd4Cre*** **mice show impaired CD8^+^ T cell responses upon LCMV infection.** (A) *Hdac1^f/f^* and *Hdac1^f/f^Cd4Cre* mice were infected i.v. with 200 pfu LCMV (Armstrong). On day 6, spleen and liver were isolated and cells were analyzed. The percentage of viral-specific CD8^+^ T cells was determined using MHC class I tetramers specific for the viral peptides gp33 (tet-gp33). Diagram shows the percentage of tet-gp33^+^ CD8^+^ T cell populations isolated from spleen and liver of *Hdac1^f/f^* and *Hdac1^f/f^Cd4Cre* mice. Mean ± SD is shown (n = 4, analyzed in 1 experiment). (B) Mice were infected as described in A. On day 6, spleens and livers were isolated and cell suspensions were re-stimulated with gp33 peptide for 5 hours. IFNγ and TNFα expression was determined by intracellular cytokine staining. The percentage of INFγ^+^ or of TNFα^+^ producing CD8^+^ T cells is shown (mean ± SEM; n = 4, analyzed in 1 experiment, except for IFNγ^+^ in the spleen where n = 3). (A, B) Statistical analysis was performed using a two-tailed non-paired Student’s t test. The P-values were defined as following: *, P<0.05; **, P<0.01; ***, P<0.001.
(TIF)

Figure S5 **Reduced CD44^hi^ CD8^+^ T cell subsets in** ***Hdac1^f/f^LckCre*** **mice in the absence of PLZF of IL-15.** (A) CD44 and CD62L expression on splenic CD8^+^ T cells from *Hdac1^f/f^* and *Hdac1^f/f^LckCre* mice that have been crossed with mice that have either wild-type (*Plzf^+/+^*, upper panels) or the mutated “luxoid” (*Plzf^lu/lu^*, lower panels) *Plzf* alleles. Data are representative of 2 mice analyzed in 2 independent experiments. (B) CD44 and CD62L expression on splenic CD8^+^ T cells from *Hdac1^f/f^* and *Hdac1^f/f^LckCre* mice that have been crossed with *Il15^+/+^* (upper panels) or *Il15^−/−^* (lower panels) mice. Data are representative of 2 mice analyzed in 2 independent experiments. (A, B) The numbers indicate the percentage of cells in the respective quadrants.
(TIF)
