# Supplementary figures and images for: HDAC1 Controls CD8+ T Cell Homeostasis and Antiviral Response
Source: PLoS One. 2014 Oct 21;9(10):e110576. doi: 10.1371/journal.pone.0110576 (PMC4204873; doi:10.1371/journal.pone.0110576)

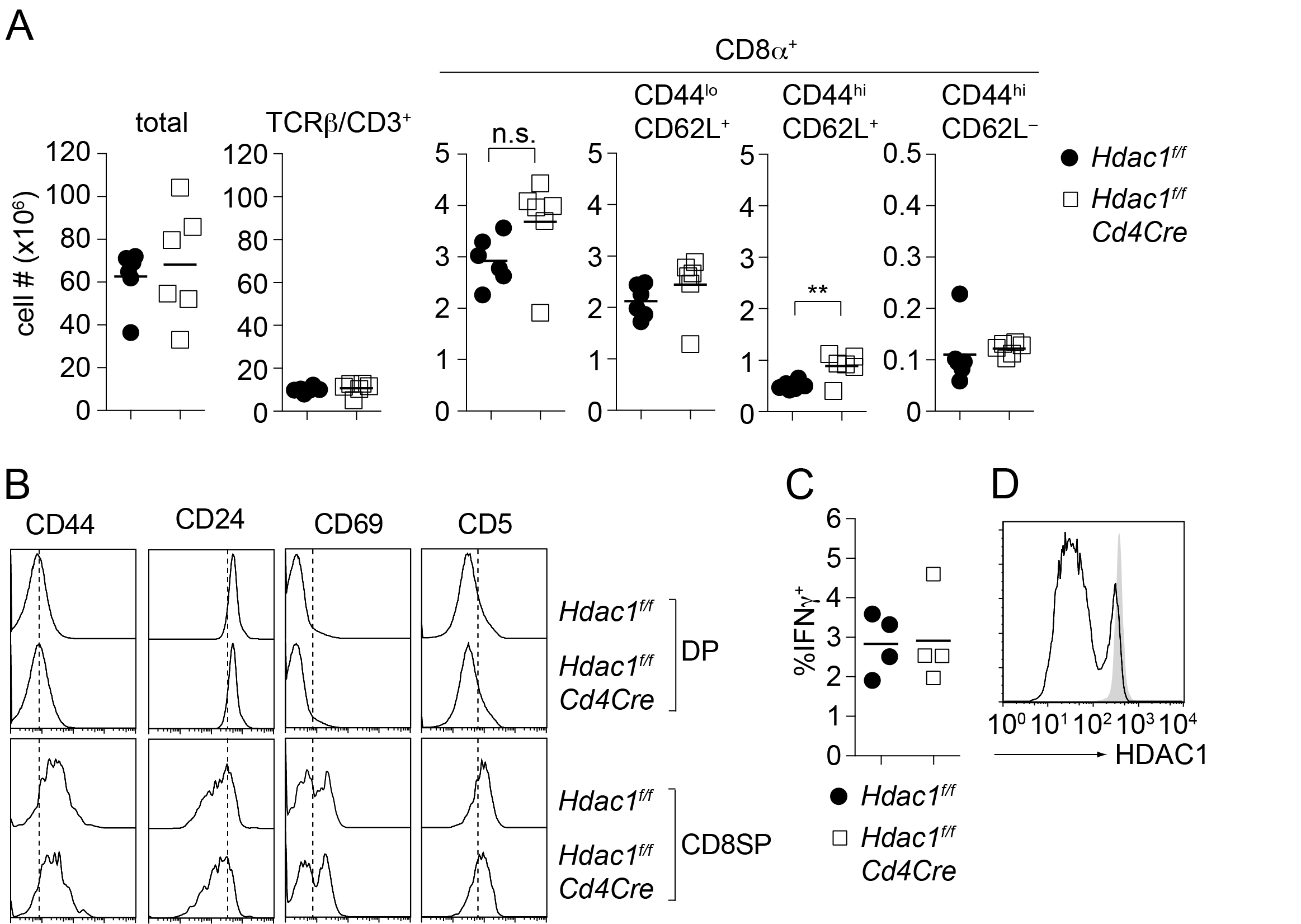

Supplement: Figure S1 — Characterization of T cell and thymocyte subsets in Hdac1f/fCd4Cre mice. (A) Diagrams showing cell numbers of Hdac1f/f and Hdac1f/fCd4Cre splenic subpopulations (mean ± SEM; n = 6, performed in 4 independent experiments). Cell numbers for total splenocytes and CD3+ T cells are in agreement with previously published data (16). (B) CD44, CD24, CD69 and CD5 expression levels on Hdac1f/f and Hdac1f/fCd4Cre CD8SP thymocytes (n = 4, performed in 2 independent experiments). (C) Thymocytes isolated from Hdac1f/f and Hdac1f/fCd4Cre mice were stimulated ex vivo with PMA/ionomycin for 4 h and IFNγ production was analyzed by intracellular cytokine staining. Mean ± SEM is shown (n = 4, performed in 2 independent experiments). Cells were gated on CD8SP thymocytes. (D) Intracellular HDAC1 levels in Hdac1f/f (solid grey) and Hdac1f/fCd4Cre (black line) CD8SP thymocytes. Data shown are representative of 2 independent experiments. (A, C) Statistical analysis was performed using a two-tailed non-paired Student’s t test. The P-values were defined as following: *, P<0.05; **, P<0.01; ***, P<0.001; n.s., not significant. (TIF) [file pone.0110576.s001.tif]

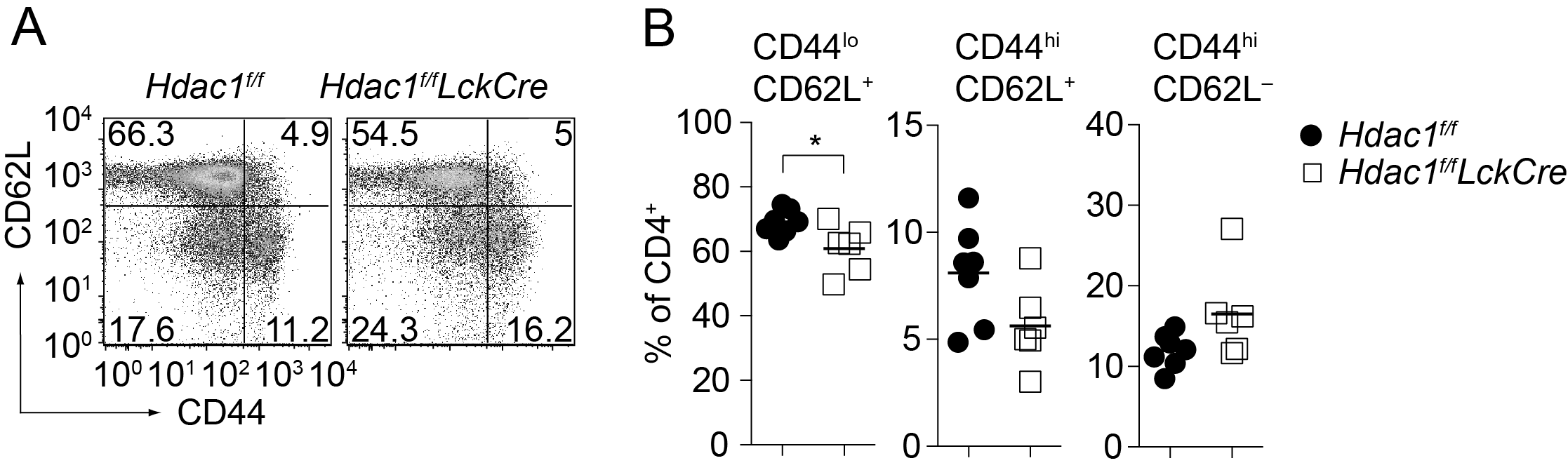

Supplement: Figure S2 — The distribution of naïve and memory CD4+ T cells in Hdac1f/fLckCre mice. (A) CD44/CD62L expression on peripheral Hdac1f/f and Hdac1f/fCd4LckCre CD4+ T cells. Numbers in the plots indicate the percentage of cells in the respective quadrants. (B) The summary of the percentage of CD44l°CD62L+, CD44hiCD62L+ and CD44hiCD62L– Hdac1f/f (n = 6) and Hdac1f/fLckCre (n = 7) CD4+ T cells is shown (mean ± SEM; performed in 4 independent experiments). Statistical analysis was performed using a two-tailed and non-paired Student’s t test. The P-values were defined as following: *, P<0.05; **, P<0.01; ***, P<0.001; n.s., not significant. (TIF) [file pone.0110576.s002.tif]

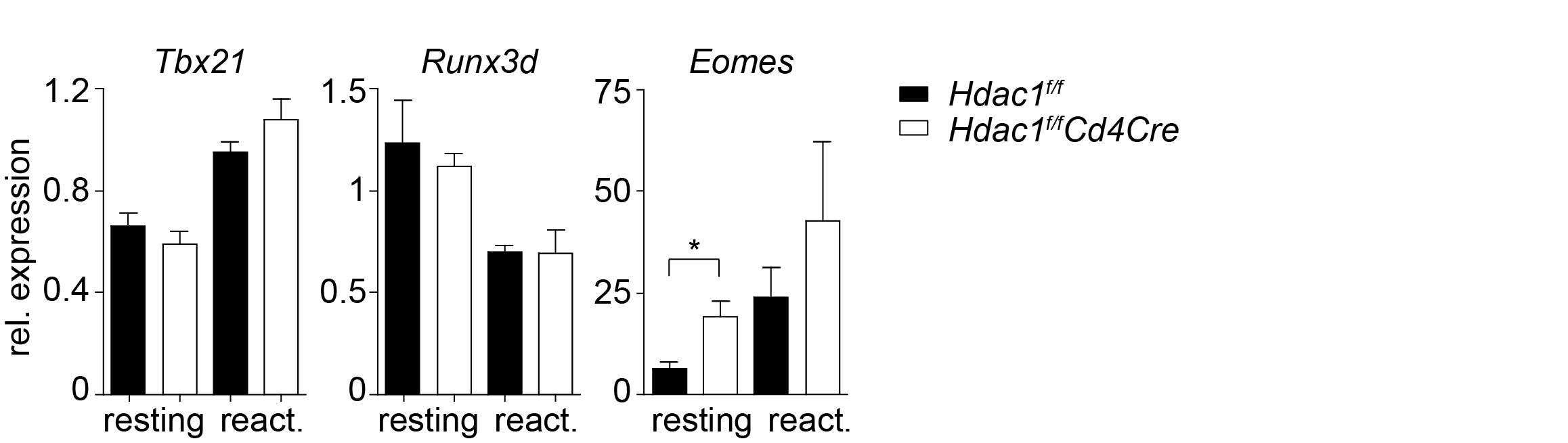

Supplement: Figure S3 — Expression of key transcription factors in Hdac1f/fCd4Cre CD8+ T cells. Naïve Hdac1f/f and Hdac1f/fCd4Cre CD8+ T cells were stimulated with anti-CD3/anti-CD28 for 48 hours. Cells were split 1∶2 on day 2, cultured for 2 additional days and re-stimulated with anti-CD3 overnight. The expression of Tbx21, Runx3d and Eomes was assessed by qRTPCR before (“resting”) and after (“react.”) overnight restimulation with anti-CD3. Expression was normalized to Hprt1 expression (mean ± SEM; n = 3; performed in 3 independent experiments). Statistical analysis was performed using a two-tailed and non-paired Student’s t test. The P-values were defined as following: *, P<0.05. (TIF) [file pone.0110576.s003.tif]

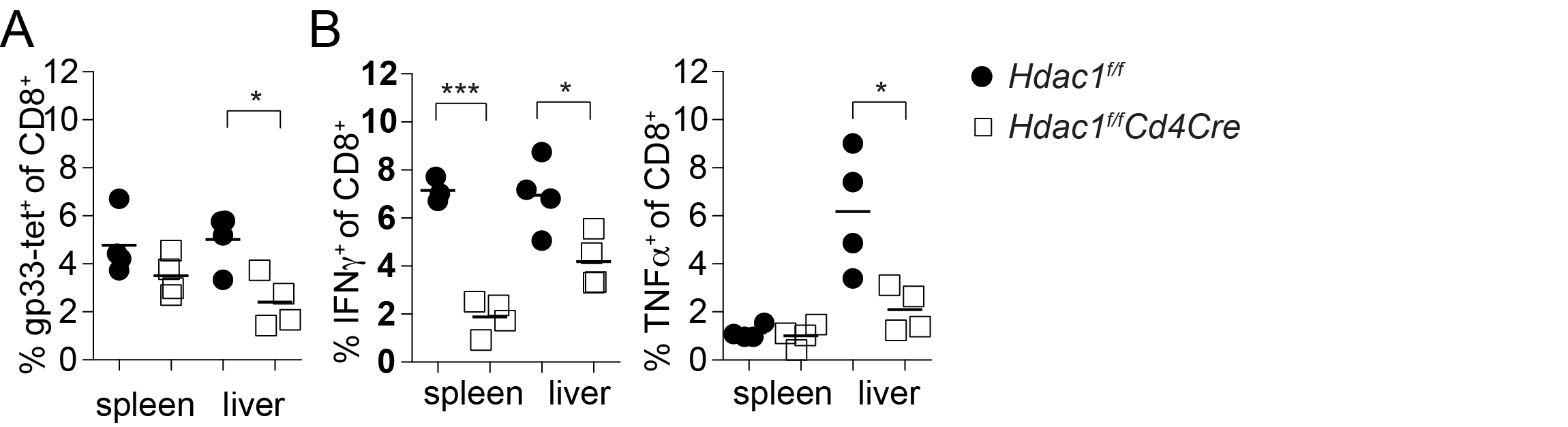

Supplement: Figure S4 — Hdac1f/fCd4Cre mice show impaired CD8+ T cell responses upon LCMV infection. (A) Hdac1f/f and Hdac1f/fCd4Cre mice were infected i.v. with 200 pfu LCMV (Armstrong). On day 6, spleen and liver were isolated and cells were analyzed. The percentage of viral-specific CD8+ T cells was determined using MHC class I tetramers specific for the viral peptides gp33 (tet-gp33). Diagram shows the percentage of tet-gp33+ CD8+ T cell populations isolated from spleen and liver of Hdac1f/f and Hdac1f/fCd4Cre mice. Mean ± SD is shown (n = 4, analyzed in 1 experiment). (B) Mice were infected as described in A. On day 6, spleens and livers were isolated and cell suspensions were re-stimulated with gp33 peptide for 5 hours. IFNγ and TNFα expression was determined by intracellular cytokine staining. The percentage of INFγ+ or of TNFα+ producing CD8+ T cells is shown (mean ± SEM; n = 4, analyzed in 1 experiment, except for IFNγ+ in the spleen where n = 3). (A, B) Statistical analysis was performed using a two-tailed non-paired Student’s t test. The P-values were defined as following: *, P<0.05; **, P<0.01; ***, P<0.001. (TIF) [file pone.0110576.s004.tif]

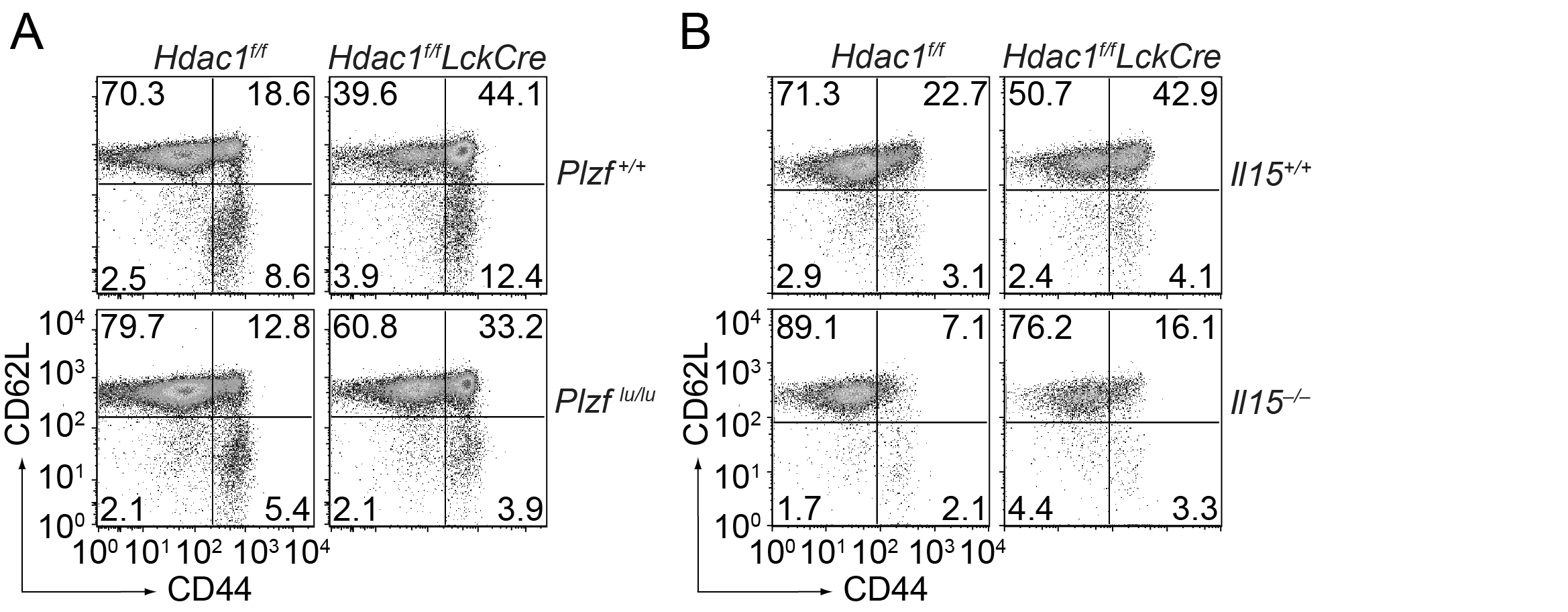

Supplement: Figure S5 — Reduced CD44hi CD8+ T cell subsets in Hdac1f/fLckCre mice in the absence of PLZF of IL-15. (A) CD44 and CD62L expression on splenic CD8+ T cells from Hdac1f/f and Hdac1f/fLckCre mice that have been crossed with mice that have either wild-type (Plzf+/+, upper panels) or the mutated “luxoid” (Plzflu/lu, lower panels) Plzf alleles. Data are representative of 2 mice analyzed in 2 independent experiments. (B) CD44 and CD62L expression on splenic CD8+ T cells from Hdac1f/f and Hdac1f/fLckCre mice that have been crossed with Il15+/+ (upper panels) or Il15−/− (lower panels) mice. Data are representative of 2 mice analyzed in 2 independent experiments. (A, B) The numbers indicate the percentage of cells in the respective quadrants. (TIF) [file pone.0110576.s005.tif]
